# The role of foreign accent and short-term exposure in speech-in-speech recognition

**DOI:** 10.3758/s13414-019-01767-8

**Published:** 2019-05-22

**Authors:** Susanne Brouwer

**Affiliations:** Radboud University, Nijmegen, The Netherlands

**Keywords:** Adaptation, Attention, Foreign accent, Short-term exposure, Speech-in-speech recognition

## Abstract

Daily speech communication often takes place in suboptimal listening conditions, in which interlocutors typically need to segregate the target signal from the background sounds. The present study investigated the influence on speech recognition of a relatively familiar foreign accent in background speech (Exp. 1) and whether short-term immediate exposure to the target talker’s voice (Exp. 2) or the background babble (Exp. 3) would either help or hinder the segregation of target from background. A total of 72 native Dutch participants were asked to listen to Dutch target sentences in the presence of Dutch or German-accented Dutch babble without (Exp. 1) or with (Exps. 2 and 3) an exposure phase. Their task was to write down what they heard. The results of Experiment 1 revealed that listeners gained a release from masking when the background speech was accented, indicating that dissimilar and less familiar signals are easier to segregate effectively. Experiment 2 demonstrated that short-term immediate exposure to the target talker had no effect on speech-in-speech recognition, whereas exposure to the background babble could hinder separating the target voice from the background speech (Exp. 3). However, this reduced release from masking only appeared in the more difficult and more familiar babble condition (Dutch in Dutch), in which the speech recognition system may have remained attuned to the babble as a potential source of communicatively relevant information. Overall, this research provides evidence that both short-term adaptation and the degrees of target–background similarity and familiarity are of importance for speech-in-speech recognition.

Speech communication rarely takes place under quiet listening conditions. Typically, interlocutors are in noisy environments in which they need to segregate the target signal from background sounds. Pollack (1975) proposed a distinction between energetic and informational masking (see also Carhart, Tillman, & Greetis, 1969; and see Kidd, Mason, Richards, Gallun, & Durlach, 2007, for a review). *Energetic* masking refers to difficulties understanding the target signal because of spectral and temporal overlap with the noise. In the case of *informational* masking, however, the target and noise may both be audible but may be difficult to separate due to linguistic, attentional, and/or other cognitive factors. Previous research has demonstrated that recognition of the target signal may be affected by the linguistic content of the background signal (e.g., Calandruccio, Brouwer, Van Engen, Bradlow, & Dhar, 2013; Garcia-Lecumberri & Cooke, 2006; Van Engen & Bradlow, 2007). That work has provided accumulating evidence that a target–background language match influences speech-in-speech recognition negatively (i.e., the *target–masker linguistic similarity hypothesis*; Brouwer, Van Engen, Calandruccio, & Bradlow, 2012). The present study aimed to test the limits of this hypothesis by examining the influences of a standard dialect of a language (i.e., Dutch) and of the same language spoken with a foreign accent (i.e., German-accented Dutch) on speech recognition. In addition, this study investigated the extent to which short-term immediate exposure to the target talker’s voice or the background babble enhances and/or deteriorates speech-from-speech segregation.

Previous work has shown several target–background variations that may improve or decrease speech-in-speech recognition. For example, a release in masking has been observed when the background language mismatches the target language, an effect that has been established for both typologically distant languages (e.g., English in Mandarin Chinese as compared to English in English; Van Engen & Bradlow, 2007) and for closely related languages (e.g., English in Dutch as compared to English in English; Brouwer et al., 2012). Moreover, Brouwer (2017) has recently extended the *target–masker linguistic similarity hypothesis* by showing that it also holds when a regional dialect (i.e., Limburgian) is paired with the standard dialect (i.e., Dutch). Regional dialects are often characterized by coherent deviations in the phonetic, phonological, phonotactic, and prosodic information found within the language (e.g., Wells, 1982). The present study, however, focused on foreign-accented speech rather than dialectal variations. In foreign-accented speech, speakers often substitute irrelevant native-language phonemes for the phonemes of the target language when the target phonemes are not found in the speaker’s mother tongue (e.g., the Dutch word *buik* [boeyk], for “belly,” is pronounced as [bɔɪk] by German speakers; Witteman, Weber, & McQueen, 2013). Native listeners thus have to deal with pronunciations that deviate from their language standards. Besides such deviations from the native phonemes, the syllable structure and prosodic patterns may also be affected by foreign-accented speech.

Since it is estimated that more than half of the world’s population speaks at least two languages (Grosjean, 2010), foreign-accented speech has become a standard listening situation. Only two studies so far have looked at whether foreign-accented speech in the background hinders speech recognition as much as does native speech in the background. Freyman, Balakrishnan, and Helfer (2001) tested native English listeners on a speech-in-speech recognition task. They were listening to English target speech in a background of either native English or Dutch-accented English. The matching condition (native English) was found to be easier than the mismatching condition (Dutch-accented English). This finding goes against the target–masker linguistic similarity hypothesis (Brouwer et al., 2012). Importantly, however, the target and background speech in that study consisted of semantically anomalous sentences, and the listeners were unfamiliar with either Dutch or a Dutch accent. These two factors could perhaps have accounted for the lack of a target–background (mis)match effect.

Similarly, Calandruccio, Dhar, and Bradlow (2010) examined the influence of native background speech (i.e., English) versus background speech with different degrees of a foreign accent on speech-in-speech recognition. They tested native English listeners on an English sentence recognition task with background speech in an accent they were rather unfamiliar with—namely, Mandarin. Contrary to Freyman et al. (2001), they used semantically meaningful sentences for both the target and background speech, and they used a more typologically distant language for the foreign-accented background babble, which they divided into three levels of intelligibility. They found that English babble provided a significantly smaller release in masking than did the three Mandarin-accented English backgrounds. These results further showed that target recognition increased when background intelligibility decreased. In other words, the accent that was the hardest to understand was the easiest to ignore. As in Freyman et al. (2001), the listeners had no knowledge of the mismatching background language (Dutch or Mandarin Chinese, respectively). It remains unclear what the effect of a foreign accent would be if listeners were relatively familiar with both the native language of the background talker (German) as well as with the talker’s accent (German-accented Dutch).

The first aim of the present study was to examine whether the target–masker linguistic similarity hypothesis also applies for native as compared to foreign-accented speech in the background. The foreign accent used in this study was reasonably familiar to the participants, and the accent was also close to Standard Dutch, because the language that influenced the accent is typologically related to Dutch. In Experiment 1, native Dutch listeners were tested on a speech-in-speech recognition task in which they listened to meaningful Dutch target sentences in the presence of either Dutch babble or German-accented Dutch babble. The participants were recruited in Nijmegen, a city in the east of the Netherlands, near the border of Germany. Quite a few German students are enrolled at Radboud University in Nijmegen (approximately 11% of the students in 2018 were German). It was therefore possible that participants in Nijmegen, in general, would be quite frequently exposed to German-accented Dutch. To test for amounts of experience with German-accented Dutch and German, a language background questionnaire was administered (see the Method section). This questionnaire revealed that the participants were relatively familiar with German-accented Dutch and with German—that is, they had monthly or somewhat less than monthly exposure both to the accent and to German. On the basis of the target–masker linguistic similarity hypothesis, it was therefore expected that the native Dutch listeners would perform better with German-accented Dutch babble than with Dutch babble.

A second, related aim of this study was to investigate whether short-term immediate exposure could improve or reduce participants’ performance on speech-in-speech recognition. To date, relatively little research has been conducted to identify which factors in speech-in-speech processing could improve through exposure. More specifically, it is somewhat unknown whether exposure has differential effects on recognizing linguistic target–background combinations. The target–masker linguistic similarity hypothesis currently does not address the idea of what adaptation can do for speech-in-speech recognition. On the basis of the results of this study, the hypothesis might be extended through an improved understanding of how exposure interacts with performance on various speech-in-speech conditions. More specifically, it was relevant to discover whether more or less similar target–masker combinations would benefit from training. The potential efficacy of training can, in particular, be relevant for individuals with hearing loss and for second language listeners. The present study focused on the effects on speech-in-speech recognition of exposure to both the target talker’s voice and the background babble.

Previous research has shown that talker information can be an important aid for listeners to recognize spoken words (e.g., Bradlow, Nygaard, & Pisoni, 1999; Mullenix, Pisoni, & Martin, 1988; Nygaard, Sommers, & Pisoni, 1994; Palmeri, Goldinger, & Pisoni, 1993). For example, Palmeri and colleagues reported faster recognition for previously presented words when the second presentation was in the same rather than a new voice, indicating that listeners can benefit from exposure to a talker. A vast amount of literature has demonstrated that adaptation to talker-specific characteristics could occur on the time scale of minutes (e.g., Eisner & McQueen, 2005; Kraljic, Brennan, & Samuel, 2008; Kraljic & Samuel, 2005; Norris, McQueen, & Cutler, 2003), or even in a few seconds (Ladefoged & Broadbent, 1957), suggesting that some form of adaptation might occur immediately.

Similar exposure effects have been found for foreign-accented speech. In particular, research has shown that exposure to foreign-accented speech can improve speech recognition (e.g., Bent & Bradlow, 2003; Clarke, 2000; Clarke & Garrett, 2004; Gass & Varonis, 1984). Gass and Varonis asked participants to transcribe sentences produced by a nonnative talker, and found that exposure to a story read by the same nonnative talker improved participants’ transcription accuracy. Clarke and Garrett investigated how much (or little) experience with a foreign accent is required for adaptation to take place. They demonstrated that less than 1 min of prior exposure to an accent is necessary and that, under certain circumstances, only two to four sentences were enough for adaptation to occur.

So far, only one study has investigated how short-term training can improve speech-from-speech segregation (Van Engen, 2012). In a two-day speech-in-noise training experiment, Van Engen examined whether native, American English participants adapted to target talkers and various noise types. They received two training sessions with feedback of about 30 min each, during which they were exposed to English target sentences presented in speech-shaped noise, in Mandarin babble, or in English babble. The results showed that listeners who received training (as compared to a no-training control group) benefited from target talker familiarity in any noise type. Moreover, training was more effective in babble than in speech-shaped noise.

In contrast to Van Engen (2012), the present study focused on how immediately adaptation could take place in a speech-in-speech recognition context, by exposing participants directly before testing. Following previous research on exposure to (foreign-accented) speech (Clarke & Garrett, 2004; Ladefoged & Broadbent, 1957), only six sentences—that is, a few seconds—were presented, to see how immediately speech-in-speech performance can be positively or negatively affected. Two different types of exposure were under investigation here. In the first type of exposure (Exp. 2), participants were exposed to six sentences spoken by the target talker prior to performing the same speech-in-speech recognition task as in Experiment 1. This exposure phase thus did not consist of any noise, as had been the case in the Van Engen study. Given the previous research (e.g., Ladefoged & Broadbent, 1957), the expectation was that exposure to the target talker’s voice would increase performance overall. However, it could also be possible that the exposure phase would not be extensive enough to establish an increase in performance (e.g., Eisner & McQueen, 2005; Kraljic et al., 2008; Kraljic & Samuel, 2005; Norris et al., 2003).

In the second type of exposure (Exp. 3), participants were exposed to the background babble, Dutch or German-accented Dutch, prior to the same speech-in-speech recognition task as in Exp. 1. The target talker’s voice was thus not presented in the exposure phase, which differed from the training in Van Engen’s (2012) study. Contrary to Experiment 2, participants should *learn* during the exposure phase *to ignore* the background babble during the test phase. In other words, they should unlearn the “incorrect” information that they have just heard. Generally, what has been found is that learning is easier than trying to explicitly unlearn something (Dougherty, 2016). The ability to learn from errors, for example, is believed to be an important mechanism for successful second language learning (e.g., Bernat & Gvozdenko, 2005). However, research has also shown that errors do not always lead to learning. For example, brain-imaging studies have suggested that people learn more from success than from failure (e.g., Histed, Pasupathy, & Miller, 2009). Given these findings, it was expected that the performance in Experiment 3 might decrease overall, as compared to Experiment 1, since attention was driven to the to-be-ignored information, which might be difficult to unlearn. Importantly, the overall approach in this study involved comparing identical trials across experiments without (Exp. 1, baseline) versus with (Exps. 2 and 3) an exposure phase.

## Method

### Participants

Seventy-two participants took part in this study. They were randomly assigned to one of the three experiments. Twenty-four native Dutch listeners participated in each experiment. None of them reported having hearing or speech impairments. Participants each filled out a questionnaire in which they had to rate their proficiency in speaking and listening in German on a 5-point Likert scale (1 = *very bad*, 5 = *very good*). Moreover, they were asked to rate their exposure to German and German-accented Dutch (1 = *never*, 2 = *less than once a month*, 3 = *monthly*, 4 = *weekly*, 5 = *daily*). Table 1 shows descriptive statistics for the participants in each experiment. Overall, the pattern shows that participants reported their proficiency in speaking German as low and their listening skills as average. Furthermore, they reported having on average monthly or less than once-a-month exposure to German or German-accented Dutch. Independent *t* tests showed that the ratings on proficiency and amount of exposure did not differ between Experiments 1 and 2 [speaking: *t*(46) = 0.153, *p* > .1; listening: *t*(46) = – 0.461, *p* > .1; German: *t*(46) = – 0.922, *p* > .1; German-accented Dutch: *t*(46) = – 1.216, *p* > .1], nor between Experiments 1 and 3 [speaking: *t*(46) = – 0.874, *p* > .1; listening: *t*(46) = – 1.109, *p* > .1; German: *t*(46) = – 1.343, *p* > .1; German-accented Dutch: *t*(46) = – 1.129, *p* > .1].

**Table 1.** Descriptive statistics of the participants in each experiment (*SD*s between parentheses)

	Exp. 1: Baseline	Exp. 2: Target	Exp. 3: Background
Gender	12 females	13 females	13 females
Mean age	24;2 (8;3)	24;4 (9;1)	27;2 (4;7)
Proficiency			
Speaking in German	2.08 (0.83)	2.04 (1.04)	2.33 (1.13)
Listening in German	2.88 (0.90)	3.00 (0.98)	3.25 (0.94)
Exposure			
German	2.17 (1.09)	2.46 (1.10)	2.58 (1.06)
German-accented Dutch	2.42 (1.28)	2.88 (1.33)	2.83 (1.27)

### Materials

In Experiments 1–3, the same stimuli were used, except that exposure stimuli were also presented in Experiments 2 and 3. The target sentences were selected from two lists (1, 7) from the revised Bamford–Kowal–Bench (Bamford & Wilson, 1979) Standard Sentence Test. Each list contained 16 meaningful sentences with three or four keywords, for a total of 50 keywords per list (e.g., *De CLOWN had een GRAPPIG GEZICHT*, “The CLOWN had a funny face”). In total, 32 sentences were used as target sentences. These sentences were translated into Dutch by a native Dutch speaker and then produced by another native female Dutch speaker (identical to the speaker in Brouwer et al., 2012).

For the Dutch background babble, 100 English meaningful sentences were taken from the Harvard/IEEE sentence lists (IEEE Subcommittee on Subjective Measurements, 1969). These sentences were translated into Dutch and produced by two native female Dutch speakers (identical to the speakers in Brouwer et al., 2012), who were different from the target speaker. For the German-accented Dutch background babble, two native female German speakers with Dutch as a second language were asked to produce the same Dutch sentences. Both speakers lived and studied in the Netherlands. On the basis of listening to three sentences from each talker, participants rated the strength of the German talkers’ accents with a score of 3.7 (*SD* = 0.61) on a 5-point Likert scale (1 = *no accent*, 5 = *very strong accent*) and an comprehensibility score of 4.29 (*SD* = 0.69; 1 = *very poor comprehensibility*, 5 = *very high comprehensibility*).

Recordings were made in a soundproof booth (22050 Hz, 24 bit). The sentences by the same talker were concatenated in Praat (Boersma, 2001), to create different one-talker tracks. Two-talker babble tracks were created by mixing the talkers of the same language into one single audio file in Audacity. Both tracks were equalized to the same root-mean-square level and the long-term average speech spectra of the two tracks were normalized, as a means of reducing unequal amounts of energetic masking between conditions.^1^

The Dutch target sentences were pseudo-randomly combined with the appropriate two-talker background speech track for a given condition in Audacity. The babble portions used for each target sentence were different. The babble came on 500 ms before and continued for 500 ms after the target sentence. The level of the target sentences was fixed at 65 dB SPL. The babble tracks were played at 68 dB SPL in order to produce a target-to-babble ratio of – 3 dB (cf. Brouwer et al., 2012; Calandruccio et al., 2013).

In Experiment 2, participants were also exposed to six additional Dutch target sentences that were used as exposure before the test phase. These sentences were spoken by the Dutch target talker and were selected (and translated) from List 2 of the Bamford–Kowal–Bench (Bamford & Wilson, 1979) Standard Sentence Test. The duration of this target exposure was 10 s. In Experiment 3, participants were exposed to Dutch babble or German-accented Dutch babble before the test phase was initiated. These exposure babble tracks were selected from the long babble tracks that were created for all experiments. The duration of each exposure babble track was identical to the duration of the target exposure in Experiment 2.

### Procedure

In Experiments 1–3, participants were seated behind a computer monitor in an experiment room. After participants had signed a consent form, written instructions were provided on the screen. The stimuli were presented with the Presentation software (version 18.0; Neurobehavioral Systems, Inc., Berkeley, CA, www.neurobs.com) and played diotically over noise-cancelling headphones. Each experimental session lasted about 5–10 min.

**Fig. 1:**
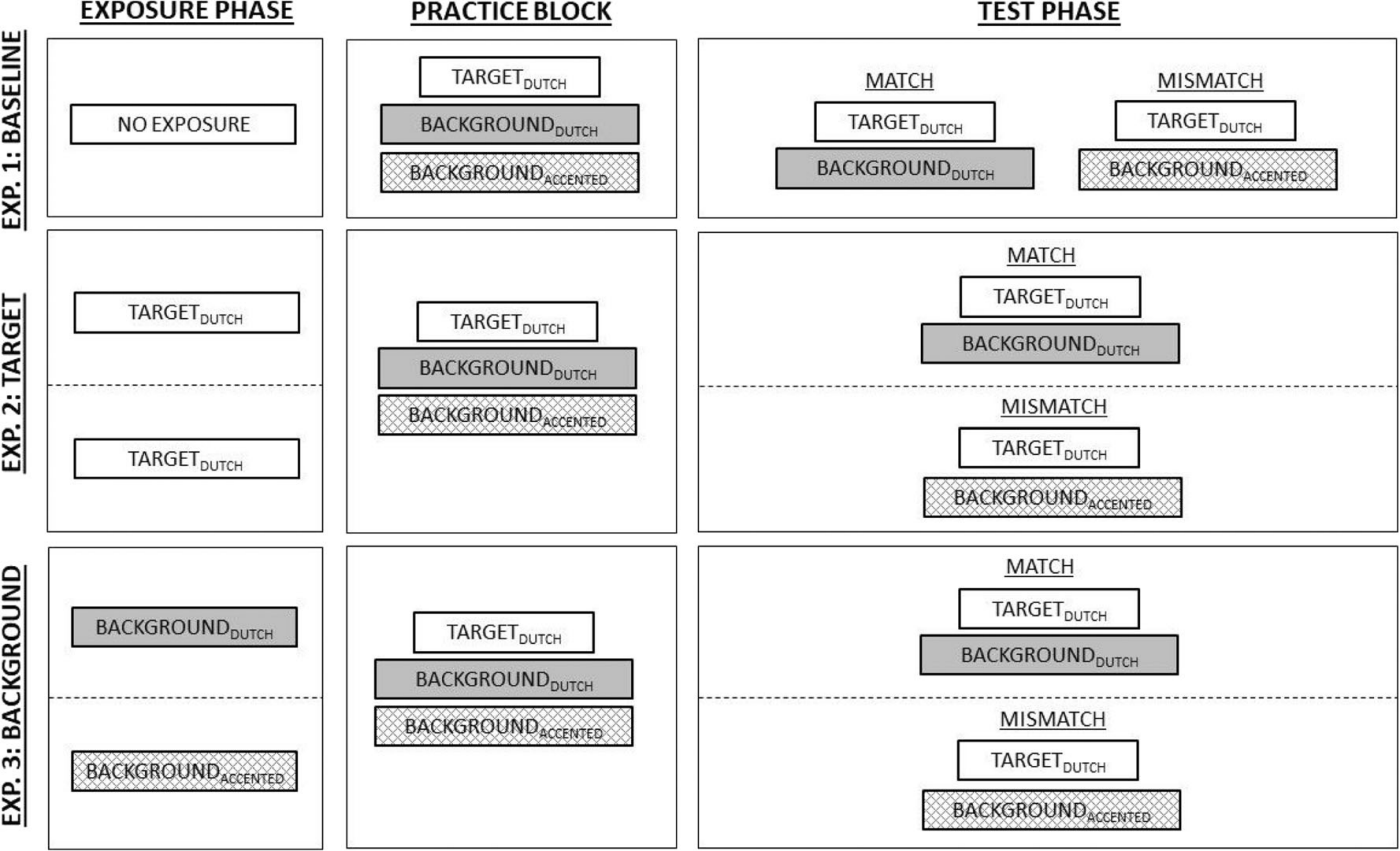
Overview of the procedures of Experiment 1 (top row), Experiment 2 (middle row), and Experiment 3 (bottom row)

In Experiment 1, participants were asked to listen to Dutch sentences spoken by a native Dutch female speaker in the presence of background babble. Their task was to report what they heard by typing the sentence using a keyboard. If they could not hear the entire sentence, they were asked to type in the individual words they were able to hear. They could only listen to each sentence once, and proceeded to the next trial by pressing the Enter button. No feedback was provided. Participants started with eight relatively easy trials in the practice session (played at signal-to-noise ratios of + 10, + 5, and 0 dB), to make sure the listeners knew whom (i.e., the target talker) to pay attention to and to familiarize themselves with the task. After the practice, participants were presented with 32 experimental items in two blocks, either Dutch babble or German-accented Dutch babble, of 16 sentences each. The sentences were presented in a randomized order. The order of the background babble was counterbalanced (see the top row of Fig. 1).

In Experiment 2, the procedure was almost identical to that of Experiment 1, except that participants were instructed to first listen to six Dutch sentences (exposure phase; see the middle row of Fig. 1) before each block. The instructions described that the talker of these sentences was identical to the target talker in the test phase. In the first block, which was counterbalanced for background babble, the exposure phase was followed by the practice block, in order to keep the same structure as in Experiment 1. The exposure phase in the second block was followed immediately by the test phase and was never followed by a practice block. Each exposure phase lasted 10 s in total.

In Experiment 3, the procedure was similar to that of Experiment 1, except that participants were instructed to first listen to multiple talkers who talked to each other and that this would sound like babbling (exposure phase; see the bottom row of Fig. 1). The instructions described that participants would hear Dutch sentences presented in the same type of babbling they had just been exposed to. It was emphasized that the participants’ task was to ignore this babbling. In the first block, which was counterbalanced for background babble, the exposure phase was followed by the practice block, in order to keep the same structure as in Experiments 1 and 2. As in Experiment 2, the exposure phase in the second block was followed immediately by the test phase and was never followed by a practice block. Importantly, the background babble in the exposure phase matched the background babble in the test phase. For example, when participants were exposed to Dutch background babble, they would hear this same type of babble in the test phase.

### Data analysis

The data were analyzed in R (version 3.2.2; R Core Team, 2018) using the glmer function from the lme4 package (Bates, Mächler, Bolker, & Walker, 2015). Linear mixed-effect regression model analyses (Baayen, Davidson, & Bates, 2008) were conducted, with keyword identification accuracy as the dichotomous dependent variable (1 = *correct*, 0 = *incorrect*). A logistic linking function was used to deal with the categorical nature of the dependent variable. Background babble (Dutch vs. German-accented Dutch; dummy-coded, with Dutch at the reference level) and experiment were entered as fixed effects. Two contrasts for the experiment factor were set up. Contrast 1 compared performance between Experiments 1 and 2, to investigate whether short-term exposure to the target talker’s voice could aid speech-in-speech recognition. Contrast 2 compared performance between Experiments 1 and 3, to examine whether short-term exposure to the background babble could improve speech-in-speech recognition. The full random-effect structure permitted by the design was included (Barr, Levy, Scheepers, & Tily, 2013). The random structure was simplified if there were signs of overparameterization (i.e., when the maximal model failed to converge). Simplification was first done by suppressing the correlation parameters, and significance was assessed via likelihood ratio tests. In this model, an effect of background babble would be evidence for a replication of the mismatched-language benefit (e.g., Brouwer et al., 2012; Van Engen & Bradlow, 2007).

## Results and discussion

**Fig. 2:**
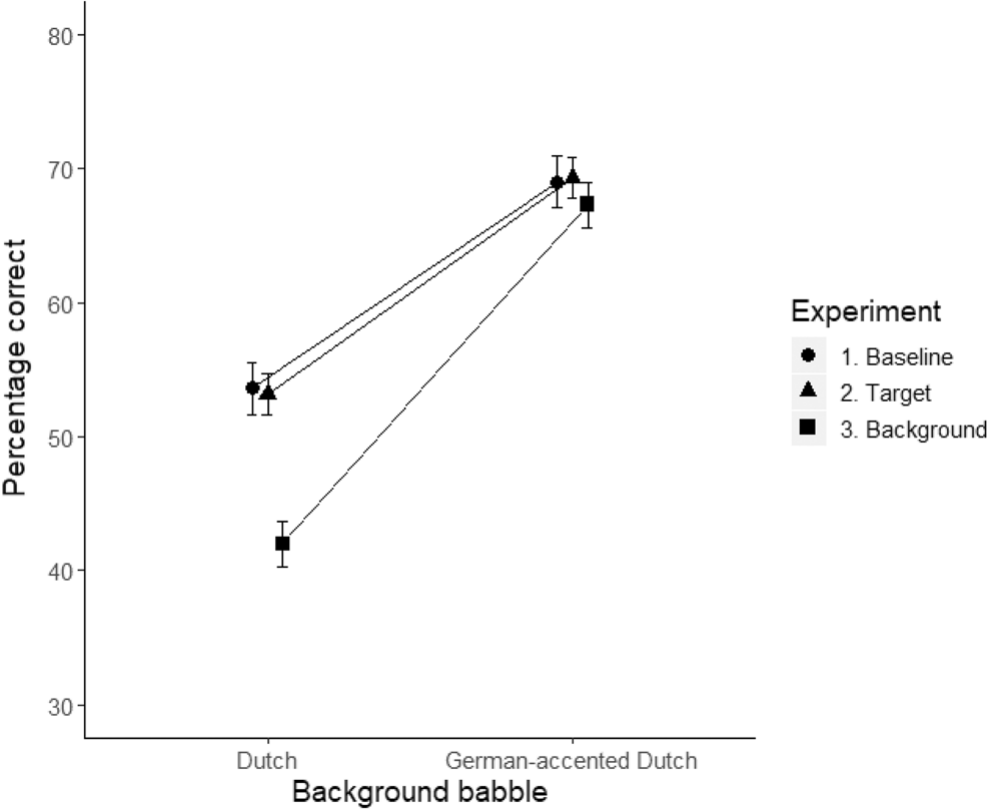
Mean intelligibility scores, in percentages of correct keyword identifications, for each background babble (Dutch, German-accented Dutch), in Experiment 1 (circles), Experiment 2 (triangles), and Experiment 3 (squares). Error bars represent standard errors

Figure 2 shows the performance of the participants in each experiment. In Experiment 1 (baseline; line with circles), recognition accuracy for Dutch sentences was on average 52.7% (*SE* = 1.47) in Dutch background babble, and 69.1% (*SE* = 1.31) in German-accented Dutch babble. In Experiment 2 (target; line with triangles), participants recognized the Dutch sentences on average with 53.2% (*SE* = 1.44) accuracy in Dutch background babble, and with 69.3% (*SE* = 1.33) accuracy in German-accented Dutch babble. In Experiment 3 (background; line with squares), the recognition accuracy for Dutch sentences was on average 42.0% (*SE* = 1.43) in Dutch background babble, and 67.3% (*SE* = 1.35) in German-accented Dutch babble.

The final model included random intercepts for participants and items. The analysis showed effects of background babble (*β* = 1.47, *SE* = 0.09, *z* value = 16.112, *p* < .0001) and experiment (Contrast 2_EXP.1 VS. 3_: *β* = – 1.05, *SE* = 0.26, *z* value = – 3.97, *p* < .0001), as well as a significant interaction between background babble and experiment (Contrast 2_EXP.1 VS. 3_: *β* = 0.95, *SE* = .18, *z* value = 5.21, *p* < .0001). All other effects were not significant (*p* > .1).

Subsequent post-hoc analyses were Tukey-adjusted for multiple comparisons using the lsmeans package (Lenth, 2016). These results are reported in Table 2. The interaction reveals that, when exposed to the background babble, listeners’ performance decreased significantly in the Dutch background condition (from *M* = 52.7 to *M* = 42.0) but not in the German-accented Dutch background condition (from *M* = 69.1 to *M* = 67.3). The significant effects will be further discussed in the next section.

**Table 2:** Post-hoc analysis comparing performance between Experiments 1 and 3 (*p* values are Tukey adjusted)

Contrasts	Estimate	*SE*	*z* Ratio	*p* Value
Exp. 1: Dutch vs. Exp. 3: Dutch	– 0.88	0.23	– 3.76	.0023
Exp. 1: German-accented vs. Exp. 3: Dutch	– 1.93	0.24	– 8.23	< .0001
Exp. 1: Dutch vs. Exp. 3: German-accented	1.07	0.23	4.62	.0001
Exp. 1: German-accented vs. Exp. 3: German-accented	0.0	0.23	0.08	1.000

## General discussion

The present study aimed to test the validity of the target–masker linguistic similarity hypothesis (Brouwer et al., 2012) by investigating the influence on speech recognition of a native language (i.e., Dutch) and the same language spoken with a foreign accent (i.e., German-accented Dutch) in the background. In addition, this study examined the extent to which short-term immediate exposure to the target talker’s voice or background babble increased or decreased speech-from-speech segregation. This will give insight into whether more or less similar target–masker combinations benefit from short-term training. Importantly, the approach in this study involved comparing identical trials across experiments with or without an exposure phase.

As predicted, the mismatching condition (i.e., German-accented Dutch babble) provided a release from masking as compared to the matching condition (i.e., Dutch babble). This result extends previous findings on the effects of background language in speech recognition and is in line with the target–masker linguistic similarity hypothesis (Brouwer et al., 2012). More specifically, the target–background (mis)match effect has previously been demonstrated for typologically dissimilar languages (e.g., Calandruccio et al., 2013; Van Engen & Bradlow, 2007), for typologically similar languages (Brouwer et al., 2012), and for standard and regional dialects of the same language (Brouwer, 2017). The present findings reveal that the target–background (mis)match effect is present not only for an unfamiliar accent of the same language as the target (Calandruccio et al., 2010), but even for a relatively familiar foreign accent of the same language. This finding goes against the results of Freyman et al. (2001), who found no effect of accentedness. But, as was outlined in the introduction, this could well have been due to the facts that Freyman et al. used semantically anomalous sentences and that their listeners were unfamiliar with the background types. The present study was conducted in an area close to the border of Germany, and participants were relatively acquainted with the foreign accent in the background babble. In a questionnaire, they reported having exposure to German and German-accented Dutch about monthly or somewhat less frequently. It would be interesting for future research to directly compare the influences on speech recognition of a less familiar to a very familiar accent.

In addition, in the present study the German talkers’ accents were judged to be fairly strong, but were at the same time considered (highly) intelligible. This contrasts with Calandruccio et al. (2010), who determined the intelligibility of the speaker on the basis of the strength of their accent, such that the high-intelligibility speaker in their study had the least noticeable foreign accent. It is possible, however, that the present accent ratings are somewhat misleading, since they were never compared to listeners’ ratings of other talkers with a foreign accent (i.e., participants only rated the two babble talkers). Future studies might further our understanding of what exactly causes speech-in-speech recognition to deteriorate, by teasing apart the strength and intelligibility of a foreign speaker’s accent. The effects of accent and intelligibility could also depend on listeners’ familiarity with the accent. It could be, for example, that a foreign accent, although strong, might become more intelligible if the listener is more familiar with either the foreign language or the foreign accent itself.

Previous research has shown that, besides familiarity, proficiency with the background language plays a role in speech recognition. For example, Brouwer et al.’s (2012) findings suggest that the degree of familiarity with the background language might only be of influence when participants are highly proficient in that language. More specifically, Dutch–English bilinguals experienced a smaller release from masking in the mismatching condition (English in Dutch) than did native English participants. This, however, was not the case when comparing native English and native Dutch participants’ performance. That is, the difference between the matching (English in English) and mismatching (English in Dutch) conditions for the English natives did not differ significantly from the difference that was found for the Dutch natives in their respective matching (Dutch in Dutch) and mismatching (Dutch in English) conditions. These findings reveal that at least a certain degree of proficiency must be reached for familiarity to impact speech-in-speech recognition. The participants in the present study reported having an intermediate level of proficiency in German. The question remains whether highly proficient Dutch–German speakers, such as simultaneous Dutch–German bilinguals, would be more negatively impacted by the mismatching condition.

The influence of familiarity with the background language raises the question of what impact short-term immediate exposure would have on speech-in-speech recognition. This was tested in Experiments 2 (target adaptation) and 3 (background adaptation). These experiments explored how exposure interacts with performance in various speech-in-speech conditions. In particular, these results aid us in discovering whether more or less similar target–background relations are positively or negatively influenced by short-term training. The results of both experiments replicated the target–background (mis)match effect found in Exp. 1, revealing the robustness of this effect. More importantly, in Experiment 2, in which listeners were exposed to a few sentences produced by the target talker, listeners’ performance did not improve as compared to those who received no exposure. A possible explanation for this lack of improvement is that the amount of exposure to the target speaker’s voice appeared to be too short to reveal an effect (cf. Van Engen, 2012). To investigate whether listeners could improve at all over time during Experiment 2, a post-hoc analysis was performed in which half (first vs. second half of the experiment) was added to the original model. The results showed a significant effect of half (*β* = 0.52, *SE* = 0.11, *z* value = 4.63, *p* < .0001), indicating that listeners performed better in the second (*M* = 64.8, *SE* = 1.34) than in the first (*M* = 57.2, *SE* = 1.48) half of Exp. 2. Notably, an effect of half was not found in Experiment 1 (*β* = – 0.55, *SE* = 0.48, *z* value = – 1.15, *p* = .25). These findings seem to confirm that listeners need more time to adapt to the target talker’s voice in order to experience beneficial effects, as opposed to in earlier work, which had demonstrated that speech adaptation may take place in as little as a few seconds or sentences (Clarke & Garrett, 2004; Ladefoged & Broadbent, 1957). An anonymous reviewer pointed out that it is also possible that the differences in results could stem from accent versus talker learning. In the previous studies, listeners were perhaps learning the accent rather than the talker, but since the listeners were already relatively familiar with the accent in the present study, the present training was ineffective. Further research will be needed to explore whether a more prolonged exposure phase would be able to elicit an advantage and uncover the boundary conditions of target talker adaptability in speech-in-speech recognition contexts.

Interestingly, the results of Experiment 3 revealed that short-term immediate exposure could have a negative impact on speech-in-speech recognition. More specifically, it was found that, after listeners were exposed to background babble, their performance decreased significantly in the Dutch background babble condition, but not in the German-accented Dutch background condition. In other words, exposure to Dutch background babble hindered separating the target from the Dutch background babble in the test phase. These findings are partly consistent with the expectations outlined in the introduction, where it was predicted that listeners would have difficulties in both conditions, because they had to explicitly unlearn the information that had been provided during the exposure phase (Dougherty, 2016).

There are two explanations of why this decrease in performance was found only for the more difficult and not for the easier condition. The first, and most likely, explanation is that ignoring familiar information (i.e., Dutch babble) may be more difficult than ignoring less familiar information (i.e., German-accented Dutch babble). Similar results have been found in previous work at a much smaller time scale (Brouwer & Bradlow, 2015). In that study, the researchers tested whether speech-in-speech recognition was influenced by variation in the target–background timing relation. More specifically, the authors investigated whether within-trial synchronous or asynchronous onset and offset of the target (i.e., English) and background (i.e., English vs. Dutch) speech influenced speech-in-speech recognition. The results showed a detrimental effect of asynchronicity for English-in-English (hard condition) but not for English-in-Dutch (easy condition) recognition. That is, exposing listeners to only 500 ms of English background babble, before the target talker’s voice was presented simultaneously with the babble, led to a decrease in performance in the English-in-English condition. In the present study, it is possible that familiarity with the Dutch background babble attracted a lot of attention to that background. The speech recognition system may therefore have remained attuned to the Dutch information stream as a potential source of communicatively relevant information. In contrast, lack of familiarity (or at least being less familiar) with the German-accented Dutch background may have turned attention away from this background. Participants could have therefore considered the German-accented Dutch background as a less informative and less relevant speech stream.

Second, it is possible that differences in the masking’s effectiveness for the individual talkers could have influenced the present results (e.g., Freyman, Helfer, & Balakrishnan, 2007). That is, the differences obtained between the Dutch and German-accented Dutch babble tracks cannot be attributed entirely to the foreign accent, since different talkers were used to produce these babble tracks. It will be difficult to address this issue in future research, since a person typically does not speak a language with and without a foreign accent at the same. And even with the same talker, confounding factors could potentially explain differences between babble streams that relate to the spectro-temporal characteristics of the particular languages.

In conclusion, the present results provide additional support for the target–masker linguistic similarity hypothesis, by showing that it also applies to language pairs produced with and without a relatively familiar foreign accent. Furthermore, short-term immediate exposure to the target talker’s voice had no effect on speech-in-speech recognition, whereas exposure to the background babble could hinder separating the target from the background speech. This diminished release from masking only appeared in the more difficult and more familiar babble condition, because the speech recognition system may have remained attuned to the this information as a potential source of communicatively relevant information. Overall, this research provides evidence that short-term adaptation and the degrees of target–background similarity and familiarity are of importance for speech-in-speech recognition. Further research will be necessary to explore how much and what type of exposure is needed in order to find beneficial adaptation effects.

## Footnotes

1 The long-term average speech spectrum (LTAS) script is available upon request. It was made by Chun Liang Chan in Praat (Boersma, 2001), who loosely based it on the script “ltasnoise.praat” by Veenker, van Delft, and Quené (see Quené & van Delft, 2010). The Praat script takes a directory of stimulus files and scales the intensity of the input sound files to the average long-term average spectrum of those sound files. It matches the duration of the longest stimulus (plus any noise padding specified in the arguments to the script) and scales it to match the average intensity of the stimuli. Two methods of spectral averaging are provided: either (1) calculating the LTAS of each file and averaging them or (2) concatenating the stimuli and breaking into equal-sized chunks and averaging the LTASs of the chunks. The script also saves the LTAS object into the output directory (along with the noise file).
